# Investigating eating architecture and the impact of the precision of recorded eating time: a cross-sectional study

**DOI:** 10.1016/j.ajcnut.2025.01.012

**Published:** 2025-01-11

**Authors:** Francisca Ibacache, Kate Northstone, Mengxuan Zou, Laura Johnson

**Affiliations:** Population Health Sciences, Bristol Medical School, Faculty of Health Sciences, University of Bristol, Bristol, United Kingdom

**Keywords:** eating architecture, eating occasion, timing precision, meal slot, meal size, meal frequency, meal timing, diet diaries, ALSPAC

## Abstract

**Background:**

The precision of recorded eating times directly affects the estimation of eating architecture, that is, size, timing, and frequency of eating. The impact of imprecise timing on estimates and associations of eating architecture with health remains unclear.

**Objectives:**

We compared eating architecture variables derived from precise with those of broad timing methods and examined associations with anthropometric-related and diet-related outcomes.

**Methods:**

Cross-sectional data came from 3-d diet diaries of 7-y-old children in the Avon Longitudinal Study of Parents and Children. We derived mean size, timing, and frequency of eating, using exact times (precise, *n* = 4855) and midpoint meal slot times (broad, *n* = 7285). Intraclass correlation coefficients (ICCs) estimated agreement between methods. Bland–Altman analysis determined mean difference and limits of agreement (LOAs). Correlations (95% CIs) estimated associations between eating architecture variables and anthropometric-related or diet-related traits.

**Results:**

Agreement varied from moderate to excellent for size (ICC: 0.75), last or first time (ICC: 0.80 or 0.58), and frequency (ICC: 0.43) of eating occasions. Broad times underestimated eating frequency (2.2 times/d; LOA: −1, 5) and overestimated size (83 g; LOA: −179, 13), last time (50 min; LOA: −142, 42), intermeal intervals (68 min; LOA: −126, −11), and eating window (49 min; LOA: −161, 63). Directions of eating architecture intercorrelations were consistent regardless of time precision but varied in magnitude, for example, larger eating occasion size correlated with lower eating frequency but was stronger with precise time (*r*_precise_ = −0.54; 95% CI: −0.56, −0.52; *r*_broad_ = −0.24; 95% CI: −0.27, −0.22). Correlations with anthropometric-related and diet-related outcomes were also directionally consistent.

**Conclusions:**

Precise timing improves the estimation of eating architecture. Differences in estimation will affect descriptions of children’s eating habits and possibly dietary guidance. However, consistent directional associations across timing methods suggest that broad times could provide a pragmatic method for investigating eating architecture associations in large samples.

## Introduction

Eating architecture is defined as the structure of eating, which includes the size, timing, and frequency of eating occasions (EOs) [1]. Single aspects of eating architecture like breakfast [2], snacking [3], late eating [4], and portion size [5] have been studied. A belief in their causal role in health is demonstrated by healthy eating guidelines recommending to eat breakfast, avoid snacking or eating late, and reduce portion size [[6], [7], [8]]. However, critics suggest that current guidelines for eating architecture are based on accepted wisdom rather than robust evidence [9]. Systematic reviews of aspects of eating architecture agree that evidence is inconsistent, low-quality, and short-term, often yielding conflicting findings in relation to health, thus limiting the ability to draw definitive conclusions and develop consistent guidelines on the matter [[2], [3], [4], [5]]. It remains unclear what the ideal size, timing, and frequency of meals and snacks should be [10].

Tools that measure habitual dietary intake [11,12] can limit the study of eating architecture at an EO level by relying on basic questions about usual eating patterns or making assumptions about fixed meals times to distinguish meals from snacks or to define breakfast, lunch, or dinner [[13], [14], [15], [16]]. Diet diaries can obtain detailed data on individual EOs over many days but they are infrequently used owing to participant and researcher burden, including time-consuming data entry and processing. Furthermore, diaries and other detailed dietary assessment tools (e.g. dietary history and 24-h recalls) vary in the precision with which time is recorded [[17], [18], [19], [20], [21], [22], [23]]. Such variation may lead to misclassification of EOs [19]. For instance, tools using broad time intervals may overlook smaller, spontaneous meals or snacks, recording them as part of a larger meal, although more precise methods capture each EO separately. These discrepancies complicate harmonizing the results across multiple studies using diverse methods.

Differences in the precision of timing between studies may underlie inconsistency in findings in systematic reviews [15,24,25]. Measurement variability is often driven by the practical limits of the data collected rather than an underlying theory supporting one method over another. Existing studies that code eating data into broader periods or a meal slot format might inadvertently combine multiple unique EOs into 1 meal [26], which can, for example, obscure distinctions between meals and snacks. Broader periods containing multiple EOs could result in overestimation of the true size of an EO, underestimation of true frequency of eating, and addition of noise to the timing of eating. Furthermore, the period covered by a meal slot can be culturally specific to normal mealtimes in that population [27], so harmonizing broad periods across populations can be problematic. Finally, variation in the duration of meal slots at different times of the day could lead to differential errors in estimating eating architecture and extra bias for studying eating at certain times of day.

To address these complexities, it is essential to examine the impact of timing precision on eating architecture. Diet records with specific times of EOs alongside broader meal slots can quantify the nature of bias created by variation in the precision of EO timing. Quantifying bias could help design better data collection or coding methods, inform analyses, and strengthen the interpretation of inconsistencies in the evidence base. By enhancing the accuracy of dietary assessments, our findings could inform public health guidelines and dietary practices aimed at optimizing nutritional intake and improving health outcomes, particularly in children. Consequently, we aimed to assess the effects of timing precision on the characterization of eating architecture variables and their associations with anthropometric measurements and dietary outcomes at age 7 y.

## Methods

### Sample and study design

The Avon Longitudinal Study of Parents and Children (ALSPAC) is an ongoing population-based prospective study centered in Bristol in the Southwest of England that initially recruited 14,541 pregnant women with expected delivery date between April 1991 and December 1992. Our analysis used a cross-sectional study design within this cohort. Detailed information on the enrolment in ALSPAC can be found elsewhere [28,29]. Efforts to recruit eligible cases at age 7 y who did not enroll originally at birth, increased the total pregnancies to 15,447, resulting in 14,901 surviving children for analyses who were alive at 1 y of age. Ethical approval for the study was obtained from the ALSPAC Ethics and Law Committee and the Local Research Ethics Committees. Informed consent for the use of data collected via questionnaires and clinics was obtained from participants following the recommendations of the ALSPAC Ethics and Law Committee at the time. When the children were 7 y old, they were invited to attend a face-to-face clinic. The study website offers a searchable data dictionary with all available data details (http://www.bristol.ac.uk/alspac/researchers/our-data/).

### Dietary assessment

Dietary intake was measured using 3-d food diaries, where parents/carers were asked to record the food and drink intake of their children for 2 weekdays and 1 weekend day. They were asked to complete these before their clinic visit [30]. In the food diary, the timing of each EO, food type, the amount consumed (and leftover amounts), and preparation method were recorded. To estimate mean daily energy intake, foods and drinks from the diet records were matched to nutrient composition using UK food composition tables [30].

### Data extraction and eating architecture variables generation

When diet diaries were originally entered into an electronic database by the ALSPAC team, the timing of EOs was categorized based on 7 predefined meal slots, representing a broad definition of EO timing (Table 1). This classification approach was aligned with the standards set by the contemporary UK National Diet and Nutrition Survey (NDNS) of Young People [31], and both studies had a primary focus on the estimation of food and nutrient intake rather than eating architecture (a field of study that has since grown in interest). As part of a study of adult eating behavior and genetics, a subset (*n* = 2933) of archived diet diaries from age 7 y were retrieved and coded to explore eating architecture by inputting data on the exact time of EOs [32]. This extraction process ensured that each food entry and EO within the electronic database included both a precise and a broad time classification. Participants were dropped if precise eating times were missing or invalid (time >23:59). Four participants with partially missing data on precise eating time for some of their EOs were dropped because a direct comparison with broad time was impossible (i.e. where the number of precise EOs was less than the broad EOs). The final sample, defined as valid data, included participants with complete and clean broad and precise timing data (*n* = 4855). In this study, 2933 diaries from the final sample were initially coded based on the availability of genetic and adult eating behavior data at age 24 y [32], although the remaining 1922 diaries were randomly sampled from the remaining archive of 7285 age 7 y diet diaries [33].

**TABLE 1:** Periods adapted from ALSPAC coding documents.

Reported time of consumption in the raw data (diary)	Implicit meal slot	Meal slot time representation
Midnight to 06:59	Overnight	06:00
07:00 to 09:59	Breakfast	08:00
10:00 to 11:59	Mid-morning	11:00
12:00 to 14:29	Lunch	13:00
14.30 to 16.59	Mid-afternoon	17:00
17:00 to 19:29	Evening	20:00
19:30 to 23:59	Late evening	22:00

Abbreviation: ALSPAC, Avon Longitudinal Study of Parents and Children.

The eating architecture variables are formally defined in Table 2. The dimensions of eating size, timing, and frequency were calculated in 2 ways: precise times and meal slot–based times. For eating size, the mean weight in grams and energy content in kilocalories of an EO were calculated by summing all foods consumed in an EO then computing the mean EO size from all EOs in all recorded days available. Eating frequency was computed on a daily basis, counting the number of unique times (precise) or meal slots (broad) containing food each day and then computing the mean frequency per day over all recorded days. Timing was represented by 4 distinct variables: *1*) the time of the first EO (continuous minutes since midnight); *2*) the time of the last EO (continuous minutes since midnight); *3*) the eating window (minutes), denoting the duration between the first and last instances of eating; and *4*) the intermeal intervals (minutes). Variables 1 and 2 are reported in 24-h clock times for ease of interpretation, although variables 3 and 4 are expressed as time durations in hours.

**TABLE 2:** Summary of eating architecture variables (mean units over the 3 diet diary days).

Eating architecture dimension	Variables (unit)	Definition
Size	1) Eating size in grams (g)	Grams per EO
	2) Eating size in calories (kcal)	Calories per EO
Timing	1) First EO time (min; h)	The timing of the first EO
	2) Last EO time (min; h)	The timing of the last EO
	3) Eating period (min; h)	The time elapsed between the first and last EO in a day
	4) Intermeal interval (min; h)	Time elapsed between one EO and another
Frequency	1) Frequency (EOs/day)	The number of EOs in a day

Abbreviation: EO, eating occasion.

### Demographics and outcome measures

Demographics included age (months) at clinic attendance, sex (male/female), ethnic background (White/other than White) and socioeconomic status—represented by maternal highest education attainment, categorized into secondary education (CSE), including vocational training; ordinary level, (GCSE; compulsory examinations taken at age 16 y); advanced level (optional examinations taken at age 18 y); and university degree and higher. Anthropometric measures encompassed BMI (kg/m^2^) [34] and waist circumference (WC, measured in centimeters), whereas dietary-related variables included total energy intake (TEI, measured in kilocalories per day) and an obesogenic dietary pattern (ODP) score. The ODP is represented by a dietary pattern *z*-score (mean 0 and SD 1), derived using reduced rank regression from the intake of 42 food groups to explain variation in dietary energy density, fiber density, the proportion of energy derived from free sugar, and the proportion of energy derived from fat intake. This dietary pattern captures relevant variation in diet and has demonstrated associations with the development of obesity during childhood, adolescence, and young adults, thus justifying its classification as obesogenic [[35], [36], [37], [38]].

### Statistical analysis

Descriptive summaries of eating architecture based on precise and broad EOs include mean (SD) for continuous normally distributed variables or median (IQR) otherwise. We did not address missing data, but used complete case analysis [39], and applied a 2-tailed test in relevant analyses to allow for the possibility of associations in either direction, reflecting our hypothesis-free approach. Pearson correlations (*r*) and 95% CIs estimated associations between the eating architecture variables. Intraclass correlation coefficients (ICCs) with 95% CIs were calculated to estimate agreement between eating architecture variables computed by either a precise or a broad method. Specifically, ICC values were derived from an individual-measure, 2-factor mixed effects consistency model to assess agreement of variables between methods considering both within-individual and between-individual variances. Bland–Altman analysis [40] and corresponding plots were generated to illustrate the absolute agreement between the same variables computed by either a broad or a precise method with the mean difference, limits of agreement (LOAs) between methods, and percentage of data points that lie outside the LOA.

To further explore the nature of misclassifications observed between methods, descriptive statistics of both broad and precise measurements of EOs within designated periods, or meal slots, was examined. In addition, mean time intervals between EOs were calculated in instances where multiple precise EOs (>1) were recorded for each broad EO (meal slot). To understand the extent to which the use of broad or precise time of eating influenced the associations between eating architecture and individual characteristics, we conducted a comparative analysis of eating architecture associations with BMI, WC, TEI, and ODP using Pearson correlation coefficients (*r*) and 95% CIs. All analyses were performed in Stata (version 17.0; StataCorp).

## Results

**FIGURE 1:**
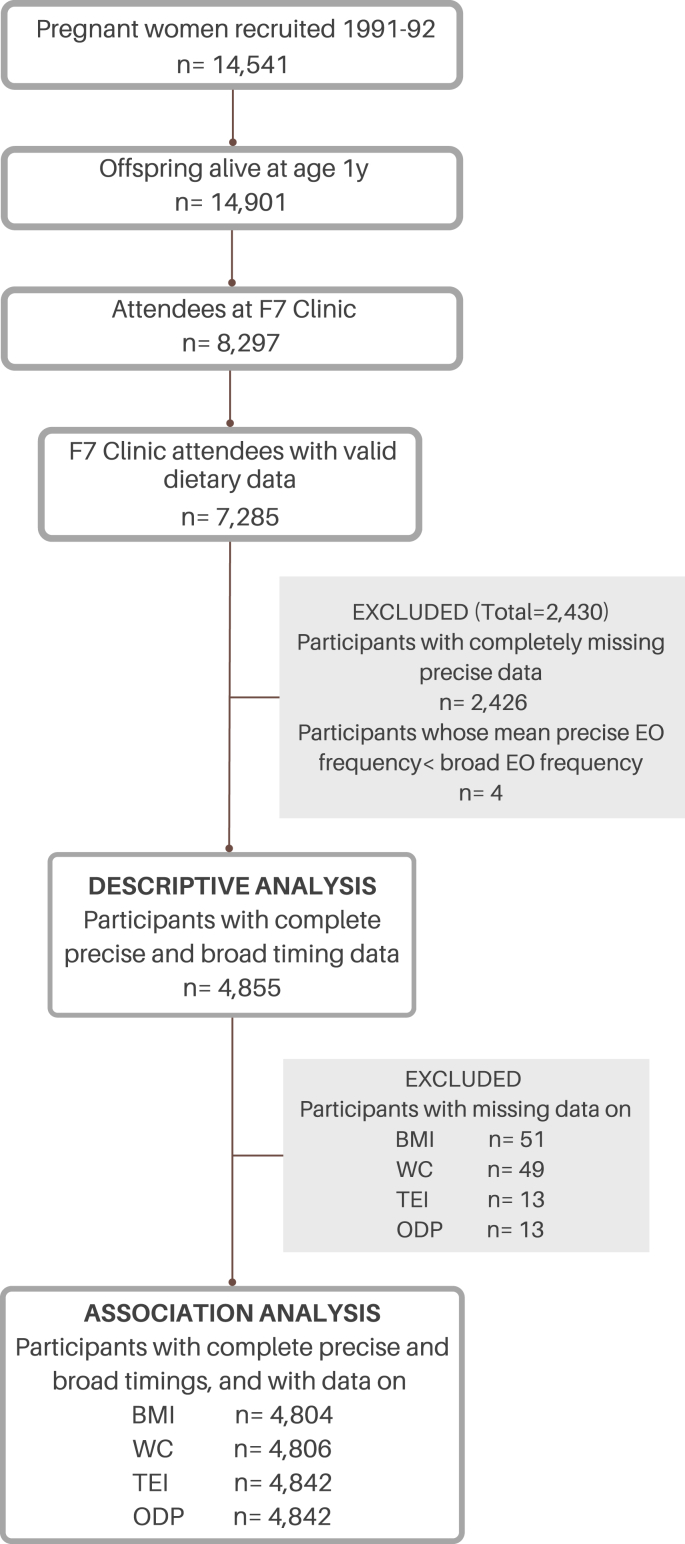
Flow diagram of the sample selection for descriptive and association analysis.

Of the 7285 children who had diet diary data, a subsample of 4868 children had their precise eating times extracted. Within this subsample, 4855 children presented valid data, constituting the final sample used for descriptive analyses. For association analyses complete dietary and anthropometric data were necessary, and sample sizes varied depending on the availability of data on the outcome variables (Figure 1). The mean age of the study sample was 7.6 y, they were mostly females (54%), and the majority of mothers were educated to O-level (roughly equivalent to grade 10 in the United States) (Table 3). Eating architecture variables are summarized in Table 3. Precise EOs were smaller (217–300 g/EO and 257–354 kcal/EO) and more frequent (7 compared with 5 EOs/d) than broad. Having precise timings did not alter the first time of eating; however, the last time was earlier (19:19 compared with 20:09), resulting in a shorter eating window (11 h 16 min compared with 12 h 5 min) and intermeal intervals (2 h 6 min compared with 3 h 14 mins) when precise time was used.

**TABLE 3:** Descriptive characteristics of ALSPAC subsample with complete broad and precise diet data and overall sample^1^.

	Subsample				Overall sample	
	*n*		Mean (SD) or (%)		*n*	Mean (SD) or (%)
Age (mo)	4842		90.6 (4.3)		7285	90.3 (3.8)
Sex						
Male	2225		46.0%		3684	50.7%
Female	2616		54.0%		3583	49.3%
Maternal education level^2^						
CSE	822		18.9%		1389	20.8%
O level	1508		34.6%		2378	35.7%
A level	1230		28.2%		1805	27.1%
Degree	800		18.3%		1092	16.4%
Ethnic background						
White	4153		96.4%		6330	96.4%
Other than White	154		3.6%		238	3.6%
TEI (kcal)	4842		1699.0 (312.1)		7267	1702.7 (314.1)
WC (cm)	4806		56.5 (5.3)		7211	56.4 (5.2)
BMI (kg/m^2^)	4804		16.3 (2.1)		7205	16.2 (2.1)
ODP (*z*-score)	4842		-0.03 (1.1)		7267	0 (1.1)

	Broad		Precise		Broad	
	*n*	Mean (SD)	*n*	Mean (SD)	*n*	Mean (SD)
Size of EO (g)	4855	300.3 (74.8)	4855	217.4 (64.1)	7285	302.4 (75.7)
Energy intake per EO (kcal)	4855	353.9 (75.4)	4855	257.4 (71.8)	7285	352.8 (74.5)
First time of EO (h:min)	4855	08:04 (00:29)	4855	08:03 (00:40)	7285	08:04 (0:28)
Last time of EO (h:min)	4855	20:09 (01:27)	4855	19:19 (00:59)	7285	20:13 (1:24)
Eating window^3^ (h, min)	4855	12, 5 (1, 31)	4855	11, 16 (01, 6)	7285	12, 10 (1, 27)
Eating interval^3^ (h, min)	4855	3, 14 (0, 33)	4855	2, 6 (0, 37)	7285	3, 12 (0, 29)
Frequency (EO/d)	4855	4.9 (0.7)	4855	7.0 (1.8)	7285	4.9 (0.7)

Abbreviations: ALSPAC, Avon Longitudinal Study of Parents and Children; BMI, body mass index; EO, eating occasion; ODP, Obesogenic dietary pattern; TEI, total energy intake; WC, waist circumference.

1 Eating architecture variables are described by the broad and precise definition in the subsample included in the analysis and overall sample (attendees at F@7 Clinic who completed diet diaries).

2 CSE: Certificate of Secondary Education (including vocational training); O level: Ordinary level (GCSE; compulsory examinations taken at age 16 y); A level: Advanced level (optional examinations sat at the age of 18 y); Degree: university degree and higher.

3 Note: eating window and eating interval represent duration in hours and minutes, not clock times.

Intercorrelations between eating architecture variables stratified by precise and broad timings are presented in Supplemental Tables 1 and 2. Larger EOs were correlated with a lower eating frequency and stopping eating earlier, whereas a higher eating frequency correlated with stopping eating later in the day, having longer eating periods, and having shorter intermeal intervals, irrespective of the precision definition applied. Although directionally consistent, correlations were stronger for precise size frequency (*r* = −0.54; 95% CI: −0.56, −0.52; compared with *r* = −0.23; 95% CI: −0.25, −0.20) and weaker for precise frequency timing (*r* = 0.48; 95% CI: 0.46, 0.50; compared with *r* = 0.65; 95% CI: 0.64, 0.67), but weak for precise and broad size timing (*r* = −0.21; 95% CI: −0.23, −0.18; compared with *r* = −0.14; 95% CI: −0.16, −0.11, respectively) associations.

The agreement of precise and broad variables was good to excellent for size and timing dimensions and moderate for frequency of EOs (Supplemental Figure 1 and Supplemental Table 3) with ICC (95% CI) for grams, last time, and frequency being 0.75 (0.74, 0.77), 0.80 (0.79, 0.81) and 0.43 (0.41, 0.46), respectively. Bland–Altman analyses revealed that when using broad timings, there was an underestimation in eating frequency by 2.2 times/d (LOA: −1, 5; 5.6%) and an overestimation of size by 83 g (LOA: −179, 13; 4.0%) and 96 kcal (LOA: −202, 9; 3.8%) per EO compared with precise timings. First EO differed by just by 1 min (LOA: −64, 61; 2.9%) between broad and precise but other timing variables were typically overestimated when using a broad method with a later last time by 50 min (LOA: −142, 42; 4.0%), and longer intermeal intervals by 68 min (LOA: −126, −11; 4.1%) and eating window by 49 min (LOA: −161, 63; 4.1%).

**FIGURE 2:**
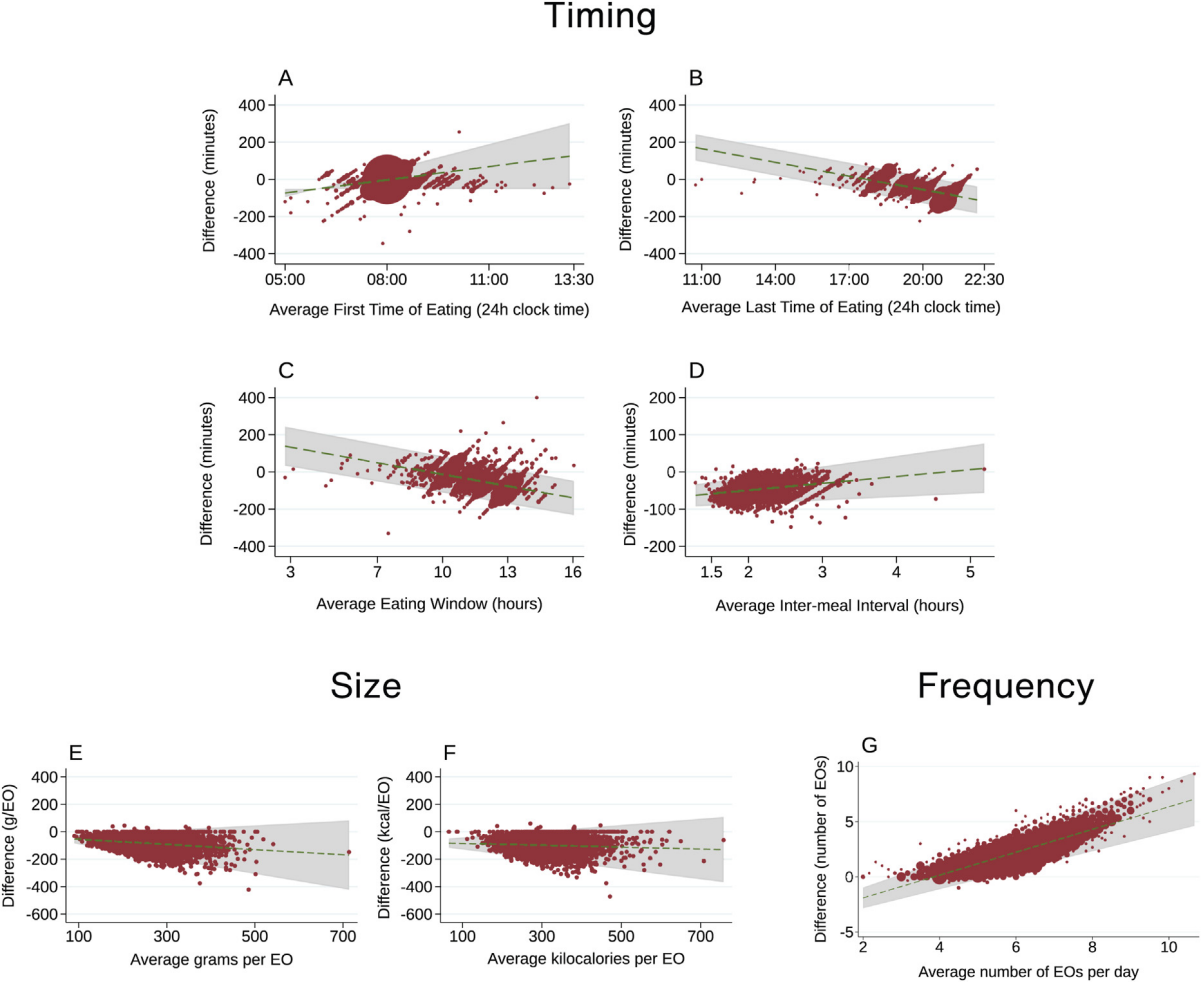
Bland–Altman plots illustrating the differences between precise and broad measurements for eating architecture variables. The plots represent timing variables—(A) mean first time of eating, (B) mean last time of eating, (C) mean eating window, and (D) mean intermeal interval; size variables—(E) mean grams per EO and (F) mean kilocalories per EO; and frequency variable—(G) mean number of EOs per day. EO, eating occasion.

There was evidence of differential disagreement, that is, bigger differences between the 2 methods at different parts of the distribution (Figure 2). Such was the case for the underestimation of eating frequency by the broad method, which was larger for increasing values of mean eating frequency. Underestimation in timing were larger for earlier initiation of eating, shorter intermeal intervals, longer eating windows, and stopping eating later. Overestimation of size was slightly greater at higher mean EO size; however, these trends were less pronounced.

**FIGURE 3:**
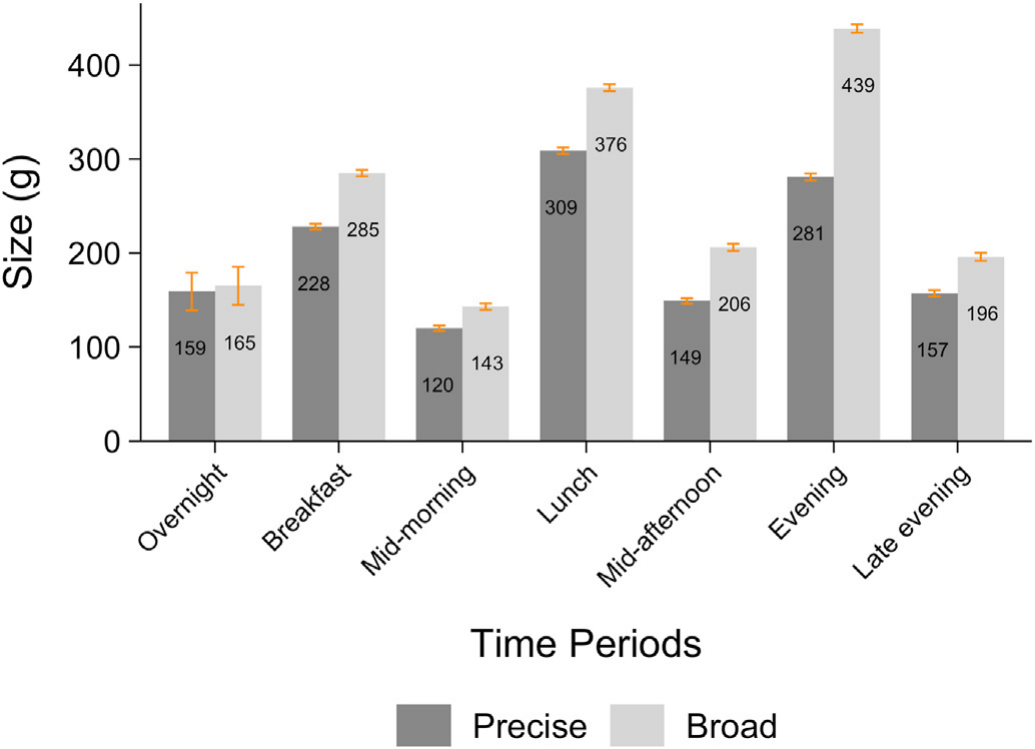
Broad and precise mean (95% CI) EO grams intake by each period (meal slots).

**FIGURE 4:**
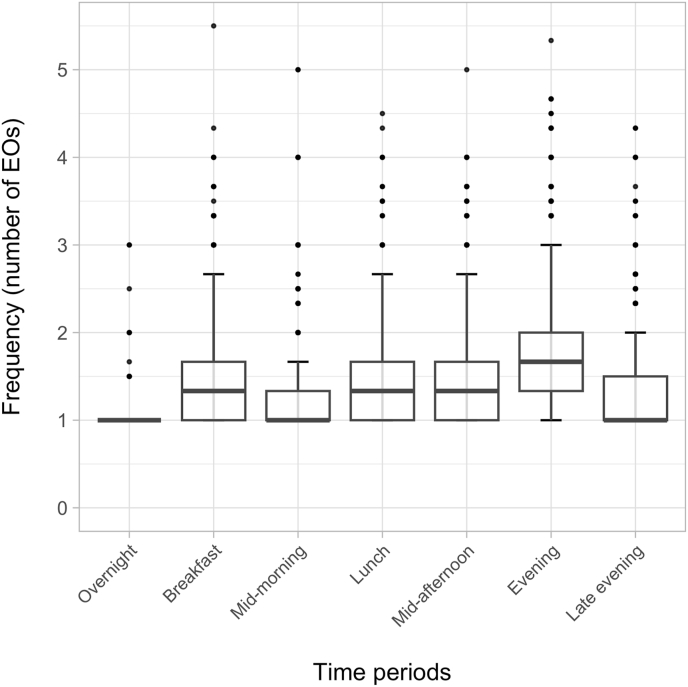
Broad and precise average EO frequency by each period (meal slots). Boxplot displaying the distribution of the frequency of EOs across periods. The box represents the IQR, with the horizontal line inside the box indicating the median. The whiskers extend to the minimum and maximum values within 1.5 times the IQR. Outliers, shown as individual points, are beyond the whiskers.

**FIGURE 5:**
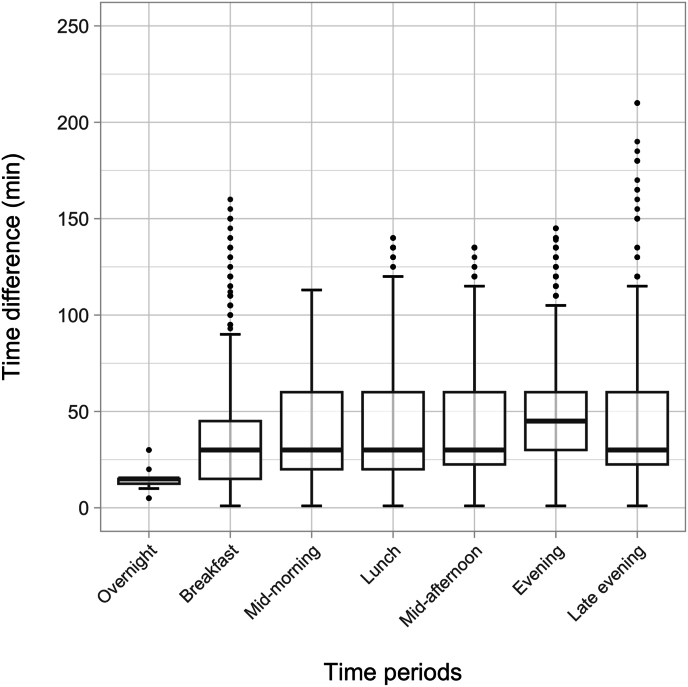
Box plot of mean time differences between individual eating occasions within a meal slot, by periods.

The underestimation of the daily number of EOs and overestimation of EO size by the broad method was further explored to understand whether meal slots at particular times of day were more likely to combine multiple EOs with each other. Figure 3 shows the mean (95% CI) of the size in grams of EOs by each method in each meal slot and confirms that the estimated amount consumed was systematically higher for the broad method, in all except the overnight meal slot. However, the biggest overestimations in EO size were observed in the evening period (158 g larger on average), followed by the lunch period (67 g larger on average). Figure 4 shows the median (IQR) of the frequency of EOs when using precise time in each broad meal slot. All meal slots sometimes combined multiple EOs, which was most likely to occur in the evening period, where a median of 1.7 (IQR 1.3, 2.0) EOs were observed (Figure 4 and Supplemental Table 4). Figure 5 displays the distribution of mean time differences between EOs that occurred within the same meal slot. Across most periods, the median time interval between EOs in the same meal slot was 30 min, ranging from as little as 5 min (10th percentile) or as big as 105 min (90th percentile). Supplemental Table 5 shows that for meal slots encompassing noon to midnight, 90% of EOs in the same meal slot are ≥15 min apart—a common cutoff used to distinguish single EOs [41], thus using a meal slot approach means that distinct EOs are being combined in error. However, a 60-min cutoff to distinguish EOs has also been used previously, and for the majority of meal slots, most of the time intervals between EOs within the same meal slot were 60 min or less (Supplemental Table 5).

**FIGURE 6:**
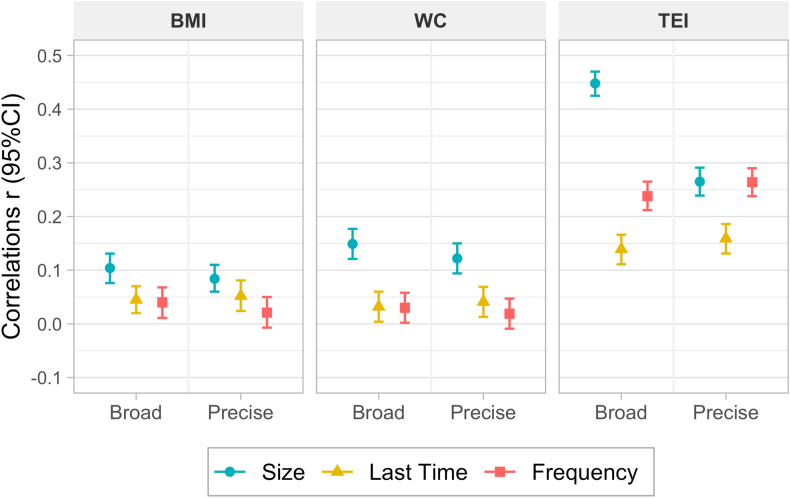
Correlations (95% CI) of eating architecture variables with total energy intake (TEI), waist circumference (WC), and BMI by precise and broad measurements. Note: Size here is represented by grams per eating occasion, and last time corresponds to the average last time of eating and represents 1 of the 4 “timing” variables created.

Correlations between the precisely and broadly estimated eating architecture variables with BMI, WC and TEI are illustrated in Figure 6. No notable differences were found in the association of size of EO between precise and broad variables in relation to BMI and WC. However, there was a greater difference between methods in the association of size with TEI, with a higher correlation between the broad size and TEI (*r* = 0.45; 95% CI: 0.43, 0.47; compared with *r* = 0.27; 95% CI: 0.24, 0.29). There were no evident differences between both approaches for frequency and timing of eating with BMI, WC, and TEI (Supplemental Table 6). Associations of eating architecture with ODP showed no differences by broad and precise (Supplemental Table 6).

## Discussion

To our knowledge, for the first time, our study has quantified the bias in eating architecture variables created by using less precise measurement of the timing of EOs and explored the impact of this bias on associations with anthropometric measurements and dietary outcomes. We found good relative agreement between EO size and timing, whereas the frequency of EOs disagreed more. Despite the disagreement observed, correlations between eating architecture variables with anthropometric-related and diet-related outcomes were directionally consistent, suggesting that use of broad timing in large samples could be a pragmatic approach to investigating eating architecture and health.

To our knowledge, no previous study has quantified the bias created by using broad compared with precise data on time of EOs. We found that the broad method underestimated eating frequency by 2 EOs/d compared with the precise method, and underestimation was accentuated for individuals eating more often. EOs with distinct precise times but within the same meal slot were combined in error leading to the underestimate of eating frequency. Multiple EOs within the same meal slot were more likely to occur in the evening, thus the last eating time varied more when precise time was used. Irrespective of precision, the biggest EOs occurred in the evening, followed by lunch and then breakfast, which is in line with previous reports of NDNS surveys on children and adolescents [42]. It is possible that smaller snacks are being consumed after dinner [43], but owing to the broader period, these snacks are being combined with the dinner EO when using a broad meal slot, potentially contributing to a perceived larger size of evening EOs. The implication of the underestimation of eating frequency we observed, specifically in the evening, could help explain why greater snacking is not consistently associated with health if some, but not all, studies combine postdinner snacks with dinner if using broader periods.

By analyzing timing structure according to broad or precise methods, we provide insight into the EOs that would be omitted when using a broader approach. When multiple EOs were combined together within a meal slot in our data, the median time interval between them was ∼30 min across most meal slots. Diverse criteria have been applied to define a unique EO, ranging from 15-min to 60-min intervals [26]. In our study, only 10% of precise EOs occurring within the same meal slot, were ≤15 min apart, suggesting that 90% of the time unique EOs are erroneously combined. Similarly, Kant et al. [44] reported that only 0.05% of EO intervals were under 15 min (*n* = 15,341 adults), whereas Leech et al. [26] concluded that an optimal EO definition involves a 15-min separation between EOs [26]. Our study suggests that using intervals of ≥15 min ensures that 90% of EOs are treated as separate events.

We saw that smaller EOs were associated with a higher frequency of eating, regardless of the precision that time was recorded at. Correlations we observed were consistent in direction and strength with other studies defining EO as energy intake within a 60-min period (*r* = −0.49) [45] or energy consumed at a unique time (*r* = −0.55) [21]. Olea López and Johnson [45] used a food-based classification to differentiate meal from snack-like EOs and reported a stronger correlation between overall intake frequency and smaller snack size (*r* = −0.21) than that with meal size (*r* = −0.01). Therefore, higher eating frequencies are likely associated more with the addition of smaller snacks, which will lower the mean EO size, rather than a reduction in meal size per se. Furthermore, larger EOs correlated with longer intermeal intervals, potentially suggesting a bidirectional compensatory effect between meal size and timing of subsequent meals [46,47]. A systematic review of experimental studies [48] highlighted that shorter intermeal intervals (<30 min) strongly influence energy compensation following a preload and thus smaller subsequent meal sizes, providing evidence that humans have robust compensatory responses over short time scales. The dynamic relationship between intermeal intervals and compensatory responses on the size of EOs adds complexity to understanding how meal size and timing interplay in dietary behaviors.

The varying durations of meal slot periods creates a differential risk of combining foods with disparate nutritional profiles into a single EO by time of day. When broader time intervals are used to define a single EO, smaller, energy-dense EOs [49,50] might be grouped together with a larger, more nutritive main meal. Combinations of EOs like this may serve to mask associations with health by mixing potentially problematic EOs, that is, energy-dense snacks, with putatively healthier ones, that is, meals. This inference is reinforced by correlations with the ODP score, indicating that larger EO sizes correspond to lower ODP scores (i.e. less obesogenic pattern of food intake). This negative association is observed with size in grams but is positive for size in kilocalories, emphasizing the importance of considering the energy density of EOs. Thus, larger overall EO sizes may reflect more meal compared with less snack eating patterns. Distinguishing between larger and smaller EOs with a more precise time may be valuable for characterizing the distinct role of meals and snacks in health.

Our findings on the association between EO size and BMI, WC, and TEI are consistent with some previous studies. For example, using precise timing, Syrad et al. [21] found that larger meal sizes (using precise timing) were associated with higher TEI, weight gain, and overweight in children. However, evidence on eating architecture and obesity-related traits in children and adolescents remains mixed, with some studies reporting little association [[51], [52], [53], [54], [55]], although others have observed benefits of practices like restricted eating windows and lower meal frequency on BMI and WC [[56], [57], [58]]. Although this study used BMI and WC as continuous variables for preliminary analyses, findings are comparable with those of studies using threshold-based measures, as continuous metrics still allow for alignment in directionality with these associations [59]. Associations between data-driven eating architecture patterns and health may also be affected by the differences in timing precision. Both Horn et al. [60] and Aqeel et al. [61] conducted cluster analyses in adults to characterize these patterns. Horn et al. used the percentage of TEI from six 4-h intervals, whereas Aqeel et al. used total kilocalories consumed in twenty-four 1-h intervals. Both identified frequent-eating patterns, but only Aqeel et al. observed associations with higher BMI and WC, whereas Horn et al. found no health outcome differences between clusters. Additionally, the frequent-eating pattern identified by Horn et al. exhibited a significantly higher mean total energy intake than the equivalent cluster in the study by Aqeel et al. (2330 compared with <1200 kcal, respectively). Therefore, using precise times may enable clearer distinctions between patterns in relation to health traits and help harmonize the health effects of meals and snacks. The associations presented are preliminary unadjusted cross-sectional correlations, and therefore, more comprehensive analyses addressing confounding and incorporating longitudinal data to improve causal inference should be applied to study associations between eating architecture and health.

Our study is affected by limitations common in nutritional epidemiology, such as dietary underreporting bias, although diet diaries demonstrate less underreporting compared with other tools [[62], [63], [64]]. Although underreporting is well-documented, it tends to pose relatively greater challenges in children during puberty than at younger ages [65,66]. In addition, our sample was primarily White UK children from Avon in the late 1990s, limiting generalizability. However, the cohort was broadly representative of the UK population at recruitment, with dietary intakes comparable with those in a national sample (NDNS) [67]. Furthermore, since data collection for this study occurred around 25 y ago, secular trends in eating architecture and dietary habits must be considered. For instance, literature based on United States children has shown increases in EO frequency and smaller eating intervals over time [68]. Using broad meal slots in contemporary data could lead to the aggregation of more EOs, which may result in greater differences in observed eating architecture between precise and broad definitions.

Our study is strengthened by using a combination of statistical tools to estimate relative and absolute agreement and quantify bias in eating architecture variables. This work breaks new ground by exploring eating architecture as a comprehensive concept, covering various facets of individuals’ eating structures rather than isolated elements, revealing important insight into how size, timing, and frequency relate to each other. The implications of our findings extend beyond diet diaries encompassing tools like 24-h recalls and diet histories, which may use exact times or broad periods. Consequently, our work holds broad relevance and offers insights into the applicability of these methods, aiding researchers in predicting bias in studies using less precise EO consumption data. Future studies should balance the limitations and convenience of using different time precision definitions and can draw on our work to indicate the possible biases introduced. Capturing broad time intervals is a pragmatic approach and may optimize time and resources although resulting in directionally consistent associations compared with more precise data. However, broad time data may lead to consistent underestimation or overestimation depending on the dimension of eating architecture of interest. Broad time is clearly suboptimal for distinguishing unique EOs so descriptive studies or investigations aiming to distinguish meals, and snacks would benefit from using precise data.

Eating architecture is an area of nutrition that needs better evidence. Consistent measurement and definitions, randomized trials that reflect real-world conditions, or longitudinal cohort studies with detailed dietary assessment would significantly advance our understanding. Our investigation of the impact of EO time precision will facilitate future research to inform the development of robust guidelines on the optimal size, timing, and frequency of eating.

## Author contributions

The authors’ responsibilities were as follows – LJ: designed research; FI, ZZ: conducted research (data collection); KN: provided essential materials necessary for the research; FI: analyzed data; FI, LJ: wrote the paper; FI, LJ: had primary responsibility for final content; and all authors: read and approved the final manuscript.

## Funding

The UK Medical Research Council and Wellcome (Grant ref: 217065/Z/19/Z) and the University of Bristol provide core support for ALSPAC. A comprehensive list of grants funding is available on the ALSPAC website (http://www.bristol.ac.uk/alspac/external/documents/grant-acknowledgements.pdf). This publication is the work of the authors, and FI and LJ will serve as guarantors for the contents of this paper. FI was supported by the National Agency for Research and Development (ANID)/DOCTORADO BECAS CHILE/2020 (72210214).

## Data availability

The study website contains details of all data that are available. The analytical code for the study can be found in the github repository (https://github.com/franibacache/Eating_architecture_precision).

## Conflict of interest

The authors report no conflicts of interest.
