# Supplementary material for: Investigating eating architecture and the impact of the precision of recorded eating time: a cross-sectional study
Source: Am J Clin Nutr. 2025 Jan 11;121(3):685–94. doi: 10.1016/j.ajcnut.2025.01.012 (PMC11923374; doi:10.1016/j.ajcnut.2025.01.012)
Supplement: multimedia component 1 [file mmc1.docx]

Supplementary Table S1. Correlations between eating architecture variables (n= 4855). Coefficients in the top right are between eating architecture variables from precise time. Coefficients in the bottom left are between eating architecture variables from broad times (meal slots).

|  | **Grams** | | | **Energy (kcal)** | | | **First Time** | | | **Last Time** | | | **Eating Period** | | | **Inter-meal Interval** | | | **Frequency** | | |
| --- | --- | --- | --- | --- | --- | --- | --- | --- | --- | --- | --- | --- | --- | --- | --- | --- | --- | --- | --- | --- | --- |
|  | **r** | **95% CI** | | **r** | **95% CI** | | **r** | **95% CI** | | **r** | **95% CI** | | **r** | **95% CI** | | **r** | **95% CI** | | **r** | **95% CI** | |
| **Grams** |  |  |  | 0.725 | 0.711 | 0.738 | 0.014 | -0.014 | 0.043 | -0.215 | -0.242 | -0.188 | -0.200 | -0.227 | -0.173 | 0.534 | 0.513 | 0.553 | -0.543 | -0.563 | -0.523 |
| **Energy (kcal)** | 0.596 | 0.577 | 0.614 |  |  |  | 0.028 | 0.000 | 0.056 | -0.344 | -0.369 | -0.319 | -0.323 | -0.348 | -0.298 | 0.672 | 0.656 | 0.687 | -0.689 | -0.703 | -0.674 |
| **First Time** | 0.038 | 0.010 | 0.066 | 0.058 | 0.029 | 0.086 |  |  |  | 0.143 | 0.115 | 0.170 | -0.475 | -0.497 | -0.453 | -0.053 | -0.081 | -0.025 | -0.080 | -0.108 | -0.052 |
| **Last Time** | -0.141 | -0.169 | -0.114 | -0.308 | -0.333 | -0.283 | 0.028 | 0.000 | 0.056 |  |  |  | 0.803 | 0.793 | 0.813 | -0.299 | -0.324 | -0.273 | 0.480 | 0.458 | 0.502 |
| **Eating Window** | -0.148 | -0.175 | -0.120 | -0.313 | -0.338 | -0.288 | -0.291 | -0.317 | -0.265 | 0.948 | 0.945 | 0.951 |  |  |  | -0.234 | -0.260 | -0.207 | 0.475 | 0.453 | 0.497 |
| **Inter-meal Interval** | 0.184 | 0.157 | 0.211 | 0.334 | 0.309 | 0.359 | -0.089 | -0.117 | -0.061 | -0.056 | -0.084 | -0.028 | -0.057 | -0.085 | -0.029 |  |  |  | -0.865 | -0.872 | -0.858 |
| **Frequency** | -0.239 | -0.265 | -0.212 | -0.457 | -0.479 | -0.434 | -0.208 | -0.235 | -0.181 | 0.651 | 0.635 | 0.667 | 0.690 | 0.675 | 0.704 | -0.714 | -0.727 | -0.700 |  |  |  |

CI, confidence interval.

Supplementary Table S2. Inter-correlations of precise (PD) vs broad (BD) defined eating architecture variables (n=4855).

|  | **Grams PD** | | | **Energy (kcal) PD** | | | **First Time PD** | | | **Last Time PD** | | | **Eating Window PD** | | | **Inter-meal Interval PD** | | | **Frequency PD** | | |
| --- | --- | --- | --- | --- | --- | --- | --- | --- | --- | --- | --- | --- | --- | --- | --- | --- | --- | --- | --- | --- | --- |
|  | **r** | **95% CI** | | **r** | **95% CI** | | **r** | **95% CI** | | **r** | **95% CI** | | **r** | **95% CI** | | **r** | **95% CI** | | **r** | **95% CI** | |
| **Grams BD** | 0.762 | 0.750 | 0.774 | 0.377 | 0.352 | 0.401 | -0.001 | -0.029 | 0.027 | -0.062 | -0.090 | -0.034 | -0.055 | -0.083 | -0.027 | 0.055 | 0.027 | 0.083 | -0.034 | -0.062 | -0.006 |
| **Energy (kcal) BD** | 0.469 | 0.447 | 0.490 | 0.732 | 0.719 | 0.745 | 0.013 | -0.016 | 0.041 | -0.219 | -0.245 | -0.192 | -0.202 | -0.229 | -0.175 | 0.200 | 0.173 | 0.227 | -0.195 | -0.222 | -0.168 |
| **First Time BD** | 0.010 | -0.018 | 0.038 | 0.020 | -0.008 | 0.048 | 0.612 | 0.594 | 0.630 | 0.079 | 0.051 | 0.107 | -0.298 | -0.324 | -0.272 | 0.006 | -0.022 | 0.034 | -0.089 | -0.117 | -0.061 |
| **Last Time BD** | -0.186 | -0.213 | -0.159 | -0.308 | -0.334 | -0.283 | 0.086 | 0.058 | 0.114 | 0.861 | 0.853 | 0.868 | 0.713 | 0.699 | 0.727 | -0.260 | -0.286 | -0.234 | 0.421 | 0.398 | 0.444 |
| **Eating Window BD** | -0.181 | -0.208 | -0.154 | -0.301 | -0.327 | -0.276 | -0.112 | -0.140 | -0.085 | 0.799 | 0.788 | 0.809 | 0.778 | 0.766 | 0.788 | -0.251 | -0.277 | -0.224 | 0.432 | 0.408 | 0.454 |
| **Inter-meal Interval BD** | 0.234 | 0.207 | 0.260 | 0.346 | 0.321 | 0.370 | 0.059 | 0.030 | 0.087 | -0.078 | -0.106 | -0.050 | -0.105 | -0.132 | -0.077 | 0.649 | 0.632 | 0.665 | -0.470 | -0.491 | -0.448 |
| **Frequency BD** | -0.300 | -0.325 | -0.274 | -0.459 | -0.481 | -0.437 | -0.124 | -0.151 | -0.096 | 0.563 | 0.544 | 0.582 | 0.575 | 0.556 | 0.594 | -0.608 | -0.625 | -0.590 | 0.646 | 0.629 | 0.662 |

CI, confidence interval.

Supplementary Table S3. Intraclass correlation coefficients (ICC) between precise and broad for each eating architecture variable.

| **Eating architecture variable** | **ICC** | **95% CI** | |
| --- | --- | --- | --- |
| Size |  |  |  |
| Grams | 0.75 | 0.74 | 0.77 |
| Energy (kcal) | 0.73 | 0.72 | 0.74 |
| Timing |  |  |  |
| First Time | 0.58 | 0.56 | 0.60 |
| Last Time | 0.80 | 0.79 | 0.81 |
| Window | 0.74 | 0.73 | 0.75 |
| Inter-meal Interval | 0.64 | 0.63 | 0.66 |
| Frequency | 0.43 | 0.41 | 0.46 |

CI, confidence interval.

Supplementary Table S4. Summary statistics for frequency of eating occasions by meal slot.

| **Meal slot** | **N** | **Mean** | **SE (mean)** | **Min** | **Max** | **IQR** | **p25** | **p50** | **p75** | **p99** |
| --- | --- | --- | --- | --- | --- | --- | --- | --- | --- | --- |
| **Overnight** | 194 | 1.1 | 0 | 1 | 3 | 0 | 1 | 1 | 1 | 3 |
| **Breakfast** | 4831 | 1.4 | 0 | 1 | 5.5 | 0.7 | 1 | 1.3 | 1.7 | 3 |
| **Mid-morning** | 3973 | 1.2 | 0 | 1 | 5 | 0.3 | 1 | 1 | 1.3 | 3 |
| **Lunch** | 4844 | 1.4 | 0 | 1 | 4.5 | 0.7 | 1 | 1.3 | 1.7 | 3 |
| **Mid-afternoon** | 4699 | 1.5 | 0 | 1 | 5 | 0.7 | 1 | 1.3 | 1.7 | 3 |
| **Evening** | 4836 | 1.8 | 0 | 1 | 5.3 | 0.7 | 1.3 | 1.7 | 2 | 3.7 |
| **Late evening** | 3730 | 1.3 | 0 | 1 | 4.3 | 0.5 | 1 | 1 | 1.5 | 3 |

SE, standard error; IQR, interquartile range.

Supplementary Table S5. Summary statistics for mean time difference between of eating occasions by meal slot.

| **Meal slot** | **N** | **Mean** | **SE (mean)** | **Min** | **Max** | **IQR** | **p10** | **p25** | **p50** | **p75** | **p90** | **p99** |
| --- | --- | --- | --- | --- | --- | --- | --- | --- | --- | --- | --- | --- |
| **Overnight** | 15 | 15.3 | 1.9 | 5 | 30 | 5 | 5 | 10 | 15 | 15 | 30 | 30 |
| **Breakfast** | 4282 | 34.1 | 0.4 | 1 | 160 | 30 | 10 | 15 | 30 | 45 | 60 | 120 |
| **Mid-morning** | 1353 | 39 | 0.6 | 1 | 113 | 40 | 12.5 | 20 | 30 | 60 | 75 | 100 |
| **Lunch** | 3669 | 44.1 | 0.5 | 1 | 140 | 40 | 15 | 20 | 30 | 60 | 90 | 120 |
| **Mid-afternoon** | 3974 | 40.9 | 0.4 | 1 | 135 | 37.5 | 15 | 22.5 | 30 | 60 | 75 | 120 |
| **Evening** | 7134 | 52.3 | 0.4 | 1 | 145 | 30 | 15 | 30 | 45 | 60 | 105 | 125 |
| **Late evening** | 1944 | 40.8 | 0.7 | 1 | 210 | 37.5 | 15 | 22.5 | 30 | 60 | 80 | 150 |

SE, standard error; IQR, interquartile range.

Supplementary Table S6. Correlations (95% CI) of eating architecture variables with total energy intake (TEI), waist circumference (WC), body mass index (BMI) and obesogenic dietary pattern (ODP) by precise and broad measurements.

|  | | **N** | **Size (g)** | | | **Energy intake (kcal)** | | | **First time (min)** | | | **Last time (min)** | | | **Window (min)** | | | **Interval (min)** | | | **Frequency (EO/day)** | | |
| --- | --- | --- | --- | --- | --- | --- | --- | --- | --- | --- | --- | --- | --- | --- | --- | --- | --- | --- | --- | --- | --- | --- | --- |
|  |  |  | **r** | **95% CI** | | **r** | **95% CI** | | **r** | **95% CI** | | **r** | **95% CI** | | **r** | **95% CI** | | **r** | **95% CI** | | **r** | **95% CI** | |
| **Body mass index (kg/m^2^)** | Precise | 4804 | 0.08 | 0.06 | 0.11 | 0.02 | -0.01 | 0.05 | 0.02 | -0.01 | 0.05 | 0.05 | 0.02 | 0.08 | 0.04 | 0.01 | 0.06 | -0.01 | -0.04 | 0.02 | 0.02 | -0.01 | 0.05 |
|  | Broad | 4804 | 0.10 | 0.08 | 0.13 | 0.03 | 0.01 | 0.06 | 0.02 | -0.01 | 0.05 | 0.05 | 0.02 | 0.07 | 0.04 | 0.01 | 0.07 | -0.01 | -0.04 | 0.02 | 0.04 | 0.01 | 0.07 |
| **Waist circumference (cm)** | Precise | 4806 | 0.12 | 0.09 | 0.15 | 0.06 | 0.03 | 0.09 | 0.00 | -0.02 | 0.03 | 0.04 | 0.01 | 0.07 | 0.03 | 0.01 | 0.06 | 0.00 | -0.03 | 0.02 | 0.02 | -0.01 | 0.05 |
|  | Broad | 4806 | 0.15 | 0.12 | 0.18 | 0.08 | 0.05 | 0.11 | 0.01 | -0.02 | 0.03 | 0.03 | 0.00 | 0.06 | 0.03 | 0.00 | 0.06 | -0.01 | -0.04 | 0.02 | 0.03 | 0.00 | 0.06 |
| **Total energy intake (kcal)** | Precise | 4842 | 0.27 | 0.24 | 0.29 | 0.42 | 0.40 | 0.44 | -0.08 | -0.10 | -0.05 | 0.16 | 0.13 | 0.19 | 0.19 | 0.16 | 0.21 | -0.23 | -0.26 | -0.21 | 0.26 | 0.24 | 0.29 |
|  | Broad | 4842 | 0.45 | 0.43 | 0.47 | 0.70 | 0.69 | 0.72 | -0.08 | -0.11 | -0.05 | 0.14 | 0.11 | 0.17 | 0.16 | 0.13 | 0.19 | -0.18 | -0.20 | -0.15 | 0.24 | 0.21 | 0.27 |
| **ODP (z-score)** | Precise | 4842 | -0.22 | -0.24 | -0.19 | 0.04 | 0.01 | 0.07 | 0.03 | 0.00 | 0.06 | 0.10 | 0.07 | 0.13 | 0.07 | 0.04 | 0.10 | -0.05 | -0.08 | -0.03 | 0.07 | 0.04 | 0.10 |
|  | Broad | 4842 | -0.21 | -0.23 | -0.18 | 0.11 | 0.08 | 0.13 | 0.03 | 0.00 | 0.05 | 0.09 | 0.06 | 0.11 | 0.07 | 0.05 | 0.10 | 0.01 | -0.02 | 0.04 | 0.04 | 0.01 | 0.07 |

EO, eating occasion; CI, confidence interval.


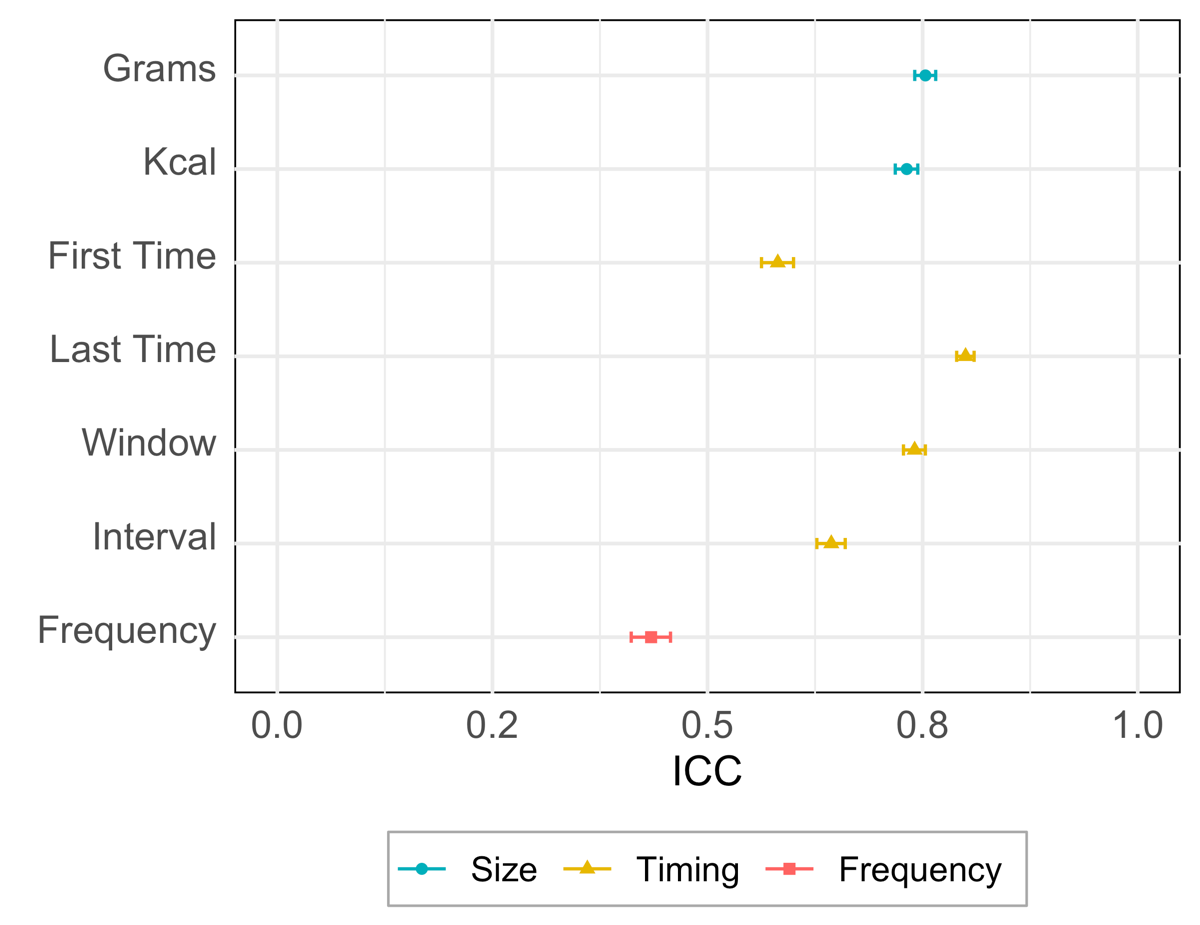


Supplementary Figure S1. Intraclass correlation coefficients (ICC) illustrating the agreement between precise and broad for each eating architecture variable.
